# The First Report of the Aphid Genus *Macromyzus* (Hemiptera: Aphididae) from Laos, with a Description of a New Species and Its Taxonomic Position

**DOI:** 10.3390/insects15121015

**Published:** 2024-12-22

**Authors:** Minho Lee, Mariusz Kanturski, Chitpasong Santammavong, Seunghwan Lee

**Affiliations:** 1 Laboratory of Insect Biosystematics, Department of Agriculture Biotechnology, Seoul National University, Seoul 151-921, Republic of Korea; v2minmin@snu.ac.kr; 2 Research Institute of Agriculture and Life Sciences, Seoul National University, Seoul 151-921, Republic of Korea; 3 Institute of Biology, Biotechnology and Environmental Protection, Faculty of Natural Sciences, University of Silesia, Bankowa 9, 40-007 Katowice, Poland; mariusz.kanturski@us.edu.pl; 4 Department of Forestry, Ministry of Agriculture and Forestry, Vientiane, 2932, Laos; chitpasong2013@yahoo.com

**Keywords:** aphids, Hemiptera, Macrosiphini, morphology, new species, SEM, sensilla

## Abstract

In this paper, the aphid genus *Macromyzus* Takahashi, 1960 is reported for the first time from Laos. A detailed description of the apterous viviparous female of a new species, *Macromyzus* (*Macromyzus*) *diplazius* sp. nov. associated with *Diplazium esculentum* is provided. For the first time, we conduct scanning electron microscopy (SEM) to examine the morphology and sensilla (antennae, mouthparts, and legs) of the representative of this genus. Furthermore, we update the taxonomic definition of the subgenus *Macromyzus* based on morphological characteristics, discuss the taxonomic position of the new species, and provide an identification key to all known *Macromyzus* species.

## Simple Summary

This study reports for the first time the genus *Macromyzus* (Aphididae: Aphidinae: Macrosiphini) from Laos. A new species, *Macromyzus* (*Macromyzus*) *diplazius* sp. nov. is described based on the apterous viviparous female. Using scanning electron microscopy, we conducted a detailed examination of the morphology and sensory structures of this genus for the first time. Additionally, the taxonomic definition of the subgenus *Macromyzus* is updated, and the taxonomic position of the newly identified species is discussed.

## 1. Introduction

The aphid genus *Macromyzus* Takahashi, 1960 is a small genus of the tribe Macrosiphini (Hemiptera: Aphididae: Aphidinae) distributed in southeastern and eastern Asia [1,2]. *Macromyzus* species exhibit strong host specificity for ferns [3]. Morphologically, this genus is characterized by a reticulated or wrinkled body surface with 2–10 pigmented tubercles at the base of the dorsal setae on each abdominal tergite, developed antennal tubercles, siphunculi with subapical zone of reticulation, and a short and triangular cauda [1,3,4]. Takahashi [5] established the genus *Macromyzus*, designating *Myzus woodwardiae* Takahashi, 1921 as the type species, which feeds on the undersides of the fronds of *Woodwardia* sp. (Blechnaceae) and *Polystichum* sp. (Dryopteridaceae) in Taiwan Island [1,4].

Historically, this genus has been the focus of debate concerning its taxonomic definition [2,3,6,7,8,9]. Takahashi [9] cautiously incorporated *Myzus polypodicola* Takahashi, 1963 into the genus *Macromyzus*, despite recognizing significant distinctions from *Macromyzus woodwardiae*. However, Ghosh et al. [7] found *Macromyzus polypodicola* (Takahashi, 1963) to be incongruent with *Macromyzus* and established a new genus, *Macromyzella*, to accommodate this species. Subsequently, three additional species (*Macromyzus indicus* David and Narayanan, 1968; *M*. *manoji* Raha and Raychaudhuri, 1978; and *M*. *spinosus* Su and Qiao, 2010) were described under the nominotypical subgenus [2,3,10,11]. Basu [6] established a new genus, *Anthracosiphoniella* Basu, 1969, designating *Anthracosiphoniella maculatum* as the type species, based on the presence of secondary rhinaria in apterous viviparae and a 4,4,4 tarsal chaetotaxy character. However, Raychaudhuri [8] reclassified *Anthracosiphoniella* as a subgenus of *Macromyzus*, arguing that the characters proposed by Basu [6] were of limited taxonomic value and emphasizing its distinctiveness in having tubercles on the spinopleural patches of the abdomen and the absence of secondary rhinaria in apterous viviparae.

In this study, we report on *Macromyzus* from Laos for the first time, providing a description of a new species accompanied by detailed morphological illustrations. Additionally, a scanning electron microscopy (SEM) analysis of *Macromyzus* is conducted for the first time, yielding precise morphological descriptions of the sensory organs critical for understanding the phylogeny and coevolution of aphids with their host plants. We also update the taxonomic definition of the subgenus *Macromyzus* based on morphological characteristics, discuss the taxonomic position of the new species, and provide a key to all known *Macromyzus* species worldwide.

## 2. Materials and Methods

### 2.1. Collection, Light Microscopy, and Abbreviations

In March 2023, several colonies of *Macromyzus* individuals were discovered on *Diplazium esculentum* (Retz.) Sw. (Athyriaceae), thriving on limestone cliffs adjacent to a waterfall (Tiger Waterfall, 15°04′04″ N, 106°12′17″ E, altitude 1010 m and 15°04′37″ N, 106°9′51″ E, altitude 1050 m) in the deep rainforest in the Boraven high plateau within the Dong Hwa Sa National Protected Area of Laos. The aphid samples were preserved in 80% ethanol, and slide glass specimens were mounted in Canada balsam, following Blackman and Eastop’s [1] method. Measurements were taken according to Ilharco and Van Harten’s method [12], and digital images were taken using Leica DMC 5400 (Leica Z16 APO) and Leica DM 4000 B (Active Measure version 3.0.3; Mitani Co. Ltd., Japan).

The following abbreviations are used: BL—body length (from the anterior border of the head to the end of the cauda); ANT—antennae; ANT I, ANT II, ANT III, ANT IV, ANT V, ANT VIb, and PT—antennal segments I, II, III, IV, V, the base of VI, and the processus terminalis of antennomere VI, respectively; GP—genital plate; HT I—the first segment of the hind tarsus; HT II—the second segment of the hind tarsus; URS—the ultimate rostral segment (segment IV+V); BW URS—the basal width of the ultimate rostral segment; H TIB—hind tibiae length; H FEM—hind femora length; MW H TIB—the middle diameter of the hind tibia; SIPH—siphunculi; BW SIPH—the basal width of the siphunculus; DW SIPH—the distal width of the siphunculus; BW Cauda—the basal width of the cauda; ABDT I–VIII—abdominal tergites I–VIII, respectively; RH ANT III—the number of secondary rhinaria on ANT III; BD ANT III—the basal diameter of antennal segment III.

### 2.2. Scanning Electron Microscopy

The dehydration of the ethanol-preserved samples was provided by ethanol series of 80, 90, and 96% and two changes of absolute ethanol for 10 min each. From absolute alcohol, the samples were transferred to pure chloroform and stored in room temperature for 24 h. The dehydrated and cleaned specimens were dried using a Leica EM CPD 300 auto critical point dryer (Leica Microsystems, Vienna, Austria). The dry samples were mounted on aluminum stubs with double-sided adhesive carbon tape and sputter-coated with a 30 nm layer of gold in a Quorum 150 T ES Plus sputter coater (Quorum Technologies Ltd., Laughton, East Sussex, UK). The specimens were imaged by a Hitachi SU8010 field emission scanning electron microscope FESEM (Hitachi High-Technologies Corporation, Tokyo, Japan) at a 10 kV accelerating voltage with a secondary electron detector (ESD) in the SEM laboratory of the Institute of Biology, Biotechnology and Environmental Protection, University of Silesia in Katowice (Katowice, Poland).

### 2.3. Depositories

The type specimens are deposited at the College for Agriculture and Life Sciences, Seoul National University Seoul, Korea (SNU). Additionally, one paratype (slide specimen) and three paratypes (scanning electron microscopy specimens) are deposited at the Zoological Collection of the University of Silesia in Katowice, Poland (DZUS).

## 3. Results

### 3.1. Taxonomy

#### 3.1.1. *Macromyzus* (*Macromyzus*) *diplazius* sp. nov.

#### 3.1.2. Apterous Viviparous Female: Description

**Figure 1:**
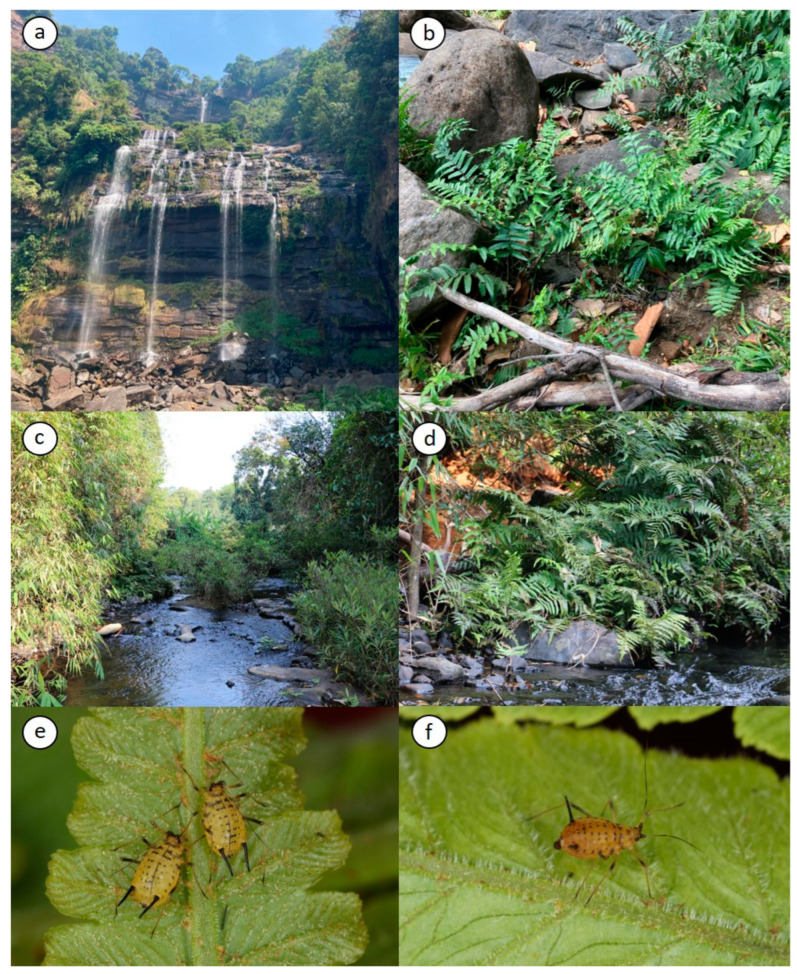
*Macromyzus* (*Macromyzus*) *diplazius* sp. nov.: (**a**,**b**) collection site (under waterfall) and host plant, (**c**,**d**) collection site (waterside) and host plant, (**e**) apterous viviparous females (body light-yellow), (**f**) apterous viviparous females (body yellow).

Color in life: body light-yellow to yellow (Figure 1e,f). Head yellow. Pronotum, metanotum, and metanotum yellow with brown band. ANT I and II dark-brown. ANT III and IV basal halves light-yellow and apical halves brown. ANT V and VI dark-brown. Legs light-brown, fore femora distal 1/4 dark-brown, middle femora dark-brown distal halves, hind femora dark-brown. Tibiae with yellow middle section and dark-brown bases and apices. SIPH, GP, and cauda dark-brown.

**Figure 2:**
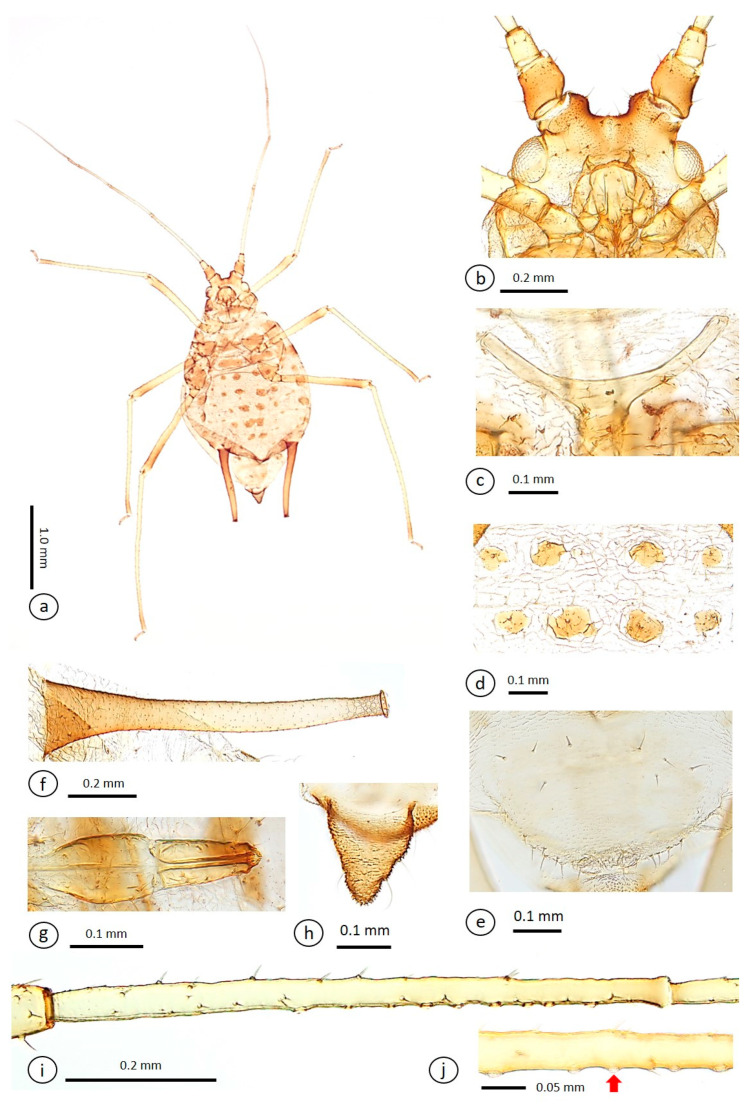
*Macromyzus* (*M*.) *diplazius* sp. nov. apterous viviparous female on mounted slide: (**a**) whole body, (**b**) head, (**c**) mesosternal furca, (**d**) dorsal patches on ABDT II–III, (**e**) GP, (**f**) SIPH, (**g**) URS, (**h**) cauda, (**i**) ANT III, (**j**) ANT III with secondary rhinaria (red arrow).

Pigmentation of mounted specimen (Figure 2a): head dark-brown. ANT I and II dark-brown. ANT III and IV basal halves light-yellow and apical halves brown. ANT V and VI dark-brown. Coxae dark-brown. Trochantera brown. Femora yellow with brown distal halves. Tibiae with yellow middle section and dark-brown bases and apices. Abdomen yellow with brown sclerites and scleroites. SIPH dark-brown with brown middle section. GP light-brown. Cauda dark-brown.

Morphometric characters: head with two pairs of cephalic ventral setae, two pairs of dorsal, and two pairs of ventral antennal tubercles setae. Dorsal setae long with incrassate apices, with one pair of dorsal setae between antennae, two pairs of dorsal setae between compound eyes, ventral setae with two pairs of ventral setae between antennae, and two pairs of ventral setae compound eyes, arranged transversely, head U-shaped between ANT (Figure 2b). ANT 1.07–1.38 × BL. ANT six-segmented. ANT III with 5–10 rounded, different-sized secondary rhinaria (Figure 2i,j). ANT IV longer than ANT V. PT 5.73–7.05 × ANT VIb. Other antennal ratios: VI:III 1.05–1.21, V:III 0.52–0.59, IV:III 0.69–0.81, PT:III 0.90–1.04, PT:IV 1.16–1.41, PT:V 1.67–1.91, and PT:VIb 5.73–7.05. Setae on ANT III 0.61–0.78 × BD ANT III. ANT bearing short, thick, and rigid setae with narrow capitate apices, ANT I–VI each with 6–9, 3–5, 24–31, 11–18, 6–11, 2–3 + 2–6 setae, respectively, apex of PT with 2 or 3 setae. Rostrum reaching hind coxae. URS 0.16–0.20 × ANT III, 0.15–0.17 × ANT VI, 0.17–0.20 × PT, 1.02–1.26 × BASE, 1.12–1.36 × HT II, and URS 1.91–2.52 × BW URS. URS wedge-shaped with 5–7 short, fine, pointed accessory setae (Figure 2g). Mesosternal furca with short stems and crescent-shaped (Figure 2c). H TIB bearing short, fine setae that are mostly slightly pointed, 0.03–0.04 mm long. H FEM 1.26–1.42 × ANT III, H TIB × 0.65–0.86 × BL. Setae on hind tibia 0.69–0.90 × MW H TIB. First tarsal chaetotaxy 4:4:3. HT II 0.14–0.16 × ANT III, 0.12–0.15 × ANT VI, 0.14–0.17 × PT and 0.84–1.08 × BASE. SIPH cylindrical, surface covered with numerous regularly distributed scales and denticles, with distinct zone of subapical reticulation, and flange (Figure 2f). The reticulated zone 0.11–0.15 × SIPH. SIPH 4.96–5.85 × cauda, 0.33–0.38 × BL, 0.96–1.23 × ANT III, and 4.23–5.71 × BW SIPH. GP with 6–8 anterior setae that are longer than the others, 13–19 posterior setae (Figure 2e). Cauda triangular to bluntly triangular, 0.98–1.27 × BW Cauda and 0.06–0.07 × BL, with 5 fine setae (Figure 2h). Anal plate transversely oval with 8–11 setae. Thorax distinctly segmented. Pronotum with wrinkles, mesonotum and metanotum with strong reticulations. Pronotum with sclerotized band. Mesonotum with 1 pair of spinal sclerites with 3–4 pairs of setae and 1 pair of marginal sclerites with 2–4 pairs of setae. Metanotum with 1 pair of sclerotized bands with 2–4 setae and 1 pair of marginal sclerites with 1–3 pair of setae. Abdomen indistinctly segmented. ABDT I–VI with strong reticulations. ABDT I–VII, each with 4, 4, 4, 4, 2, 2, and 4 spot-shaped spinal sclerites with distinct tubercles; ABDT I–V, each spinal sclerite with 1 or 2 blunt setae; ABDT VI and VII, each with 1 blunt spinal seta, respectively (Figure 2d). ABDT I–V, each with 1 pair of spot-shaped marginal sclerites with 1, 2–3, 1–4, 1–4, and 1 blunt setae, respectively. ABDT VII with 8–11 setae. ABDT VIII with 4 or 5 setae. Venter with spinulous transverse rows. Additional biometric data are presented in Table 1.

**Measurements of holotype (in mm).** BL 2.75, body width 1.76, ANT III 0.87; 0.89, ANT IV 0.66; 0.66, ANT V 0.48; 0.49, ANT VIb 0.14; 0.14, PT 0.81; 0.83, URS 0.15, H FEM 1.18; 1.22, H TIB 2.06; 2.08, HT I 0.05; 0.05, HT II 0.12; 0.13. SIPH 1.00; 1.01, BW SIPH 0.19; 0.19, DW SIPH 0.50; 0.06, Cauda 0.18, BW Cauda 0.15, BD ANT III 0.03; 0.03, MW H TIB 0.05, Cephalic setae 0.06, Setae on ANT III 0.02, Setae on ABDT I 0.04, Setae on ABDT VIII 0.06, Setae on H TIB 0.03; 0.04.

**Material examined**. Holotype: apterous viviparous female, LAOS: Paksxong (Tiger Waterfall, 15°04′04″ N, 106°12′17″ E, altitude 1010 m), 2 iii 2023, #230302-LMH-1, on *Diplazium esculentum* (Retz.) Sw. (Athyriaceae), coll. M. Lee. Paratypes: five apterous viviparous females, with the same collection data as the holotype; nine apterous viviparous females, LAOS: Paksxong (Tiger Waterfall, 15°04′37″ N, 106°9′51″ E, altitude 1050 m) (Figure 1a–d).

**Diagnosis.** The new species, *Macromyzus diplazius* sp. nov., is characterized as follows: body yellow (Figure 1e,f), ABDT I–VII each with 4, 4, 4, 4, 2, 2, and 4 dark-brown spot-shaped spinal sclerites with distinct tubercles, ABDT I–V each with 1 pair of spot-shaped marginal sclerites (Figure 2a), head U-shaped between antennae (Figure 2b), first tarsal chaetotaxy 4:4:3, number of secondary rhinaria on ANT III: 5–10 (Figure 2i,j), ABDT I–VI reticulated.

The new species is most similar to *M*. *spinosus* Su and Qiao, 2010 (original description), but there are several differences, as follows:

- Head U-shaped between antennae in the new species, while W-shaped in *M*. *spinosus*;
- Median frontal tubercle not distinct in the new species, while distinct in *M*. *spinosus*;
- Cauda/BW Cauda: 0.97–1.26 in the new species, while Cauda/BW Cauda: 1.27–1.93 in *M*. *spinosus*;
- SIPH: 0.81–1.03 mm in the new species, while SIPH: 0.59–0.61 mm in *M*. *spinosus*;
- URS/HT II: 1.12–1.36 in the new species, while URS/HT II 1.41–1.51 in *M*. *spinosus*;
- Setae on ANT III/BD ANT III: 0.61–0.78 in the new species, while setae on ANT III/BD ANT III: 0.36–0.46 in *M*. *spinosus*.

**Etymology**. The specific epithet *diplazius* is derived from the genus name of the host plant, *Diplazium*. The root *Diplaz*- originates from the Greek word diplazein, meaning “to double”, which refers to the characteristic leaf structure of the genus. The suffix -*ius* is masculine.

**Host plants**. *Diplazium esculentum* (Retz.) Sw. (Athyriaceae).

**Distribution**. Currently, it is only recorded from Laos (Paksxong).

**Biology**. This species colonizes the undersides of fern fronds and is not attended by ants.

### 3.2. Notes on SEM Morphology and Sensilla of Apterous Viviparous Female

#### 3.2.1. General Morphology and Characters

**Figure 3:**
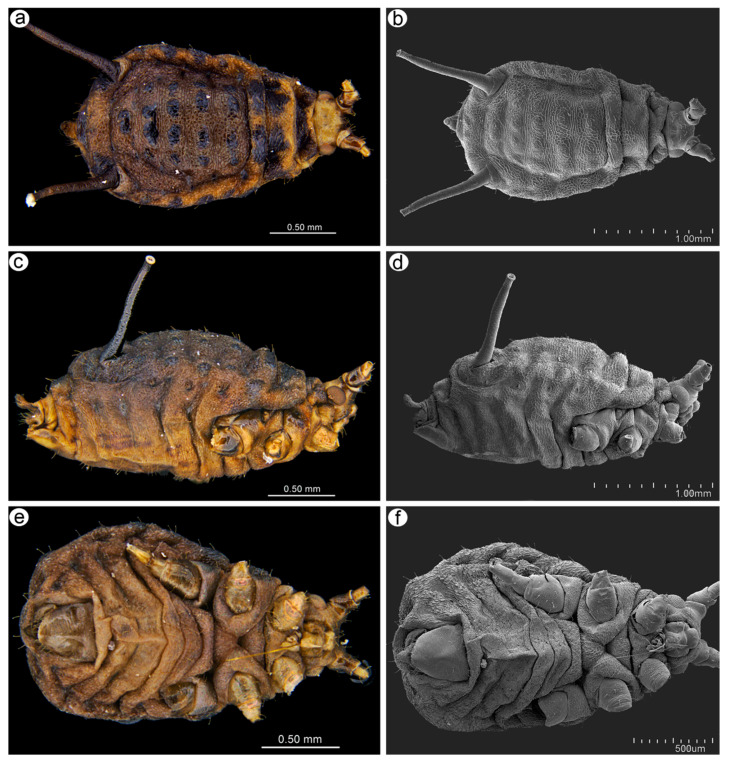
General view of *Macromyzus* (*M*.) *diplazius* sp. nov. apterous viviparous female via a stereoscope microscope (**a**,**c**,**e**) and scanning electron microscope (**b**,**d**,**f**): (**a**,**b**) dorsal side, (**c**,**d**) lateral side, (**e**,**f**) ventral side.

**Figure 4:**
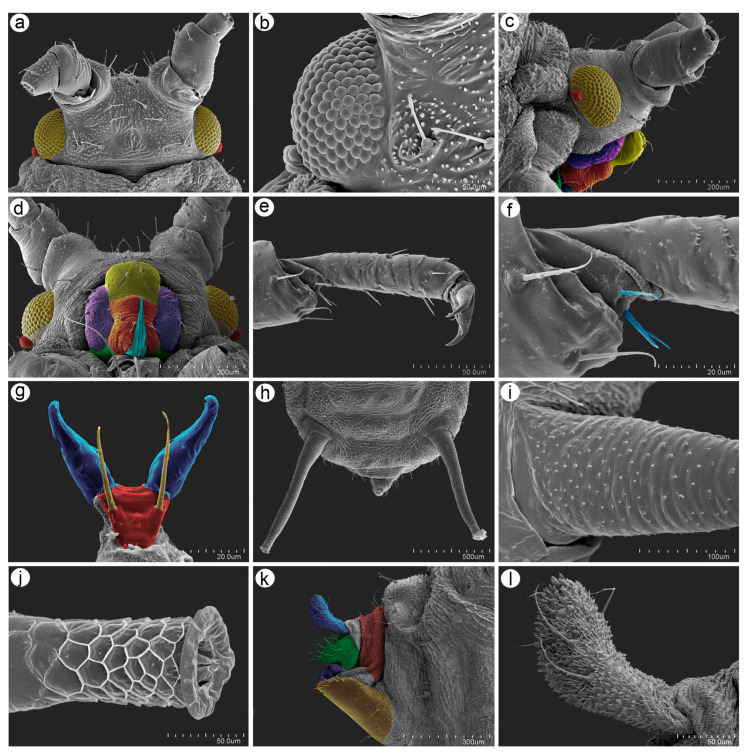
Scanning electron microscopy (SEM) images of the general characters of *Macromyzus* (*M*.) *diplazius* sp. nov.: (**a**) dorsal side of the head covered by numerous denticles with clearly visible compound eyes (yellow) and triommatidia (red), (**b**) structure of the compound eye, (**c**) lateral side of the head with compound eyes (yellow), triommatidia (red), and mouthparts: postclypeus (yellow), mandibular lamina (violet), anteclypeus (orange), and maxillary lamina (green), (**d**) ventral side of the head showing compound eyes (yellow) with triommatidia (red) and mouthparts: postclypeus (yellow), mandibular lamina (violet), anteclypeus (orange), maxillary lamina (green) and labrum (blue), (**e**) hind tarsus, (**f**) first segment of the hind tarsus, (**g**) distal end of the second segment of the hind tarsus showing claws (blue), wide pretarsus (red) and long parempodia (yellow), (**h**) end of the abdomen, (**i**) basal part of the siphunculus with numerous denticles, (**j**) distal part of the siphunculus with polygonal reticulation and well-developed flanges, (**k**) lateral side of perianal structures showing abdominal segment VIII (red), the cauda (blue), the anal plate (green), rudimentary gonapophyses (violet), and the genital plate (yellow), (**l**) cuticle structure of the cauda.

The apterous viviparous females of *M*. *diplazius* are slightly egg-shaped from the dorsal and ventral view and oval from the lateral side. The dorsal and lateral sides of the cuticle have clearly visible polygonal sculptures (Figure 3). The head is characterized by well-developed antennal tubercles and a cuticle with numerous denticles (Figure 4a). The compound eyes are well developed with many tightly adjoining ommatidia and triommatidia on the ocular tubercle (Figure 4b). From the lateral and ventral view of the head, well-developed mouthparts are visible (Figure 4c,d). The legs are of usual morphology, and the tibiae are without developed tibial attachment pads. The first segment of the tarsus is pressed to the inner side of the distal end of the tibia (Figure 4e), and three sensilla can be found on its ventral side (Figure 4f). The second segment of the tarsus is well developed with normal claws and long and tubular parempodia (empodial setae) (Figure 4e,g). The end of the abdomen is characterized by long, tubular, and slightly tapering siphunculi (Figure 4h). Their surface is covered by numerous and regularly distributed scales and denticles (Figure 4i), and their apical part is characterized by well-developed polygonal reticulation and flanges (Figure 4j). The perianal structures are characterized by a strongly bent abdominal segment VIII, curved cauda, and large genital plate (Figure 4k). The surface of the cauda is covered by a few long and pointed trichoid sensilla and numerous long microtricha (Figure 4l).

#### 3.2.2. Antennal Sensilla

**Figure 5:**
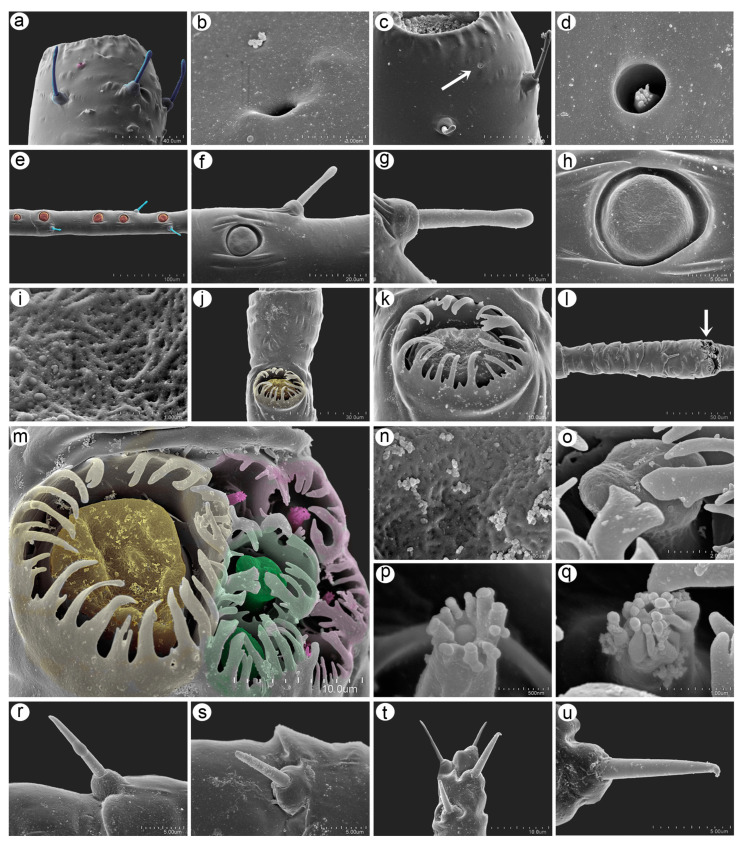
SEM images of antennal sensilla of *Macromyzus* (*M*.) *diplazius* sp. nov.: (**a**) pedicel with type I trichoid sensilla (blue) and rhinariolum (pink), (**b**) ultrastructure of rhinariolum without visible senaillum, (**c**) pedicel with rhinariolum with visible sensillum, (**d**) ultrastructure of rhinariolum opening and sensillum, (**e**) part of ANT III with type I trichoid sensilla (blue) and small multiporous placoid sensilla (orange), (**f**) structure of ANT III sensilla, (**g**) ultrastructure of type I trichoid sensillum, (**h**) ultrastructure of small multiporous placoid sensillum, (**i**) ultrastructure of porous membrane of sensillum, (**j**) distal part of ANT V with big multiporous placoid sensillum (yellow), (**k**) structure of big multiporous placoid sensillum surrounded by sclerotic collar with projections, (**l**) basal part of ANT VI with sensilla in distal part, (**m**) group of sensilla on ANT VI showing big multiporous placoid sensillum (yellow), two small multiporous placoid sensilla (green), and four sunken coeliconic sensilla of two types (pink), (**n**) ultrastructure of porous membrane of big multiporous placoid sensillum, (**o**) ultrastructure of small multiporous placoid sensillum, (**p**) type I sunken coeloconic sensillum with short projections, (**q**) ultrastructure of type II sunken coeloconic sensillum with long projections, (**r**,**s**) type II trichoid sensilla along PT, (**t**) type II trichoid sensilla on apical part of PT, (**u**) ultrastructure of type II tichoid sensillum.

Numerous antennal sensilla can be found on the antennae of the apterous viviparous female. During the SEM analyses on the pedicel, two kinds of sensilla have been found: type I trichoid sensilla and rhinariolum with a sunken coeloconic sensillum (Figure 5a). The rhinariolum may have two morphological types: type I with a smaller opening (about 1.10–1.15 μm) and a deeply lying sensillum peg (Figure 5a,b) and type II with a wider (2.30–2.40 μm) opening with a clearly visible sensillum peg. (Figure 5c). The sensillum peg is short, tapering, and with about 12–14 very short projections (Figure 5d). All remaining the segments forming the antennal flagellum bear type I trichoid sensilla (setae). On antennal segment III besides the trichoid sensilla, small multiporous placoid sensilla (secondary rhinaria) are located in one single row 218–202 μm long. The shortest distance between two sensilla is 23–24 μm, and the longest distance is 61–62 μm (Figure 5e). The sensilla diameter is 8.0–13 μm. Small multiporous placoid sensilla are rounded, are very slightly protuberant, and lie in a cuticular cavity with a very low but well-developed collar (Figure 5f,h). The porous surface of the placoid sensilla is clearly visible with two kinds of pores (rounded and narrow), 40–50 per μm^2^ (Figure 5i). Type I trichoid sensilla are tubular, thick, and rigid with flat capitate apices, are 14–18 μm in length, and lie in semicircular or slightly trapezoid sockets (Figure 5g). Near the distal end of antennal segment V, one big placoid sensillum (primary rhinarium) can be found (Figure 5j). The sensillum is rounded, is 8.0–13 μm in diameter, lies in a cuticular cavity, and is surrounded by a wide and protuberant sclerotic collar with 17–20 flat projections of different lengths (Figure 5k). On antennal segment VI, besides type I trichoid sensilla, the most numerous types of sensilla (primary rhinaria) are found, which are mostly grouped tightly on the distal part of the base of antennal segment VI (Figure 5l). In the main group, four kinds of sensilla are found: a big multiporous placoid sensillum (major rhinarium), an accessory rhinaria in the form of two small multiporous placoid sensilla, and four sunken coeloconic sensilla (two of type I and two of type II). All the sensilla are surrounded by large sclerotic collars with numerous flat projections (Figure 5m). The big multiporous placoid sensillum is rounded or oval, is 10–13 μm in diameter, and lies laterally opposite to the rest of sensilla (Figure 5m). The sensillum membrane is characterized by mostly rounded pores and some narrow ones, 22–30 pores per μm^2^ (Figure 5n). The small multiporous placoid sensilla are mushroom-shaped with rounded and flat apical parts, 3.0–4.0 μm in diameter (Figure 5o). They lie in polar positions and are located right next to the big multiporous placoid sensillum (Figure 5m). There are two types of sunken coeloconic sensilla which lie in one row on the outer lateral side of the sensilla group, opposite to the major rhinarium (Figure 5m). The sunken coeloconic sensilla are moreover in alternate positions, starting from the type I sunken coeloconic sensillum right to the big multiporous placoid sensillum and over one small multiporous placoid sensillum. The type I sunken placoid sensilla are characterized by 10–12 short projections with spherical apices which are doubled in some of the projections (Figure 5p). The type II sunken trichoid sensilla are less protuberant than the type I sensilla and are characterized by much longer projections also with spherical apices (Figure 5q). Along the antennal VI terminal process and on the very apical part, type II trichoid sensilla are found. These are short, 7.0–9.0 μm long, tubular, and rigid, with tapering and slightly flat apices (Figure 5r,s). The apical part of the terminal process bears four type II trichoid sensilla of similar length but with more pointed apices (Figure 5t,u).

#### 3.2.3. Sensilla on Mouthparts

**Figure 6:**
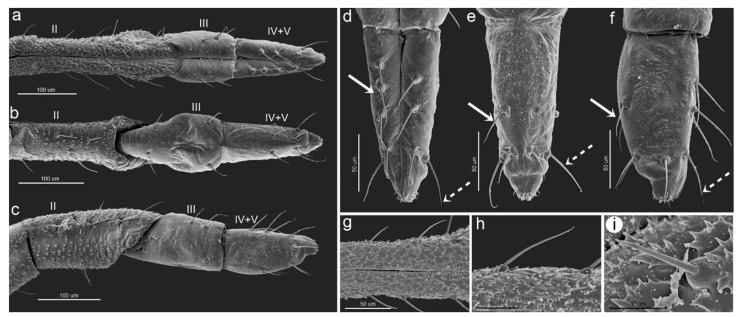
SEM images of mouthparts of *Macromyzus* (*M*.) *diplazius* sp. nov.: (**a**) ventral side of labium, (**b**) dorsal side of labium, (**c**) lateral side of labium, II—second rostral segment, III—third rostral segment, IV+V—fourth and fifth rostral segments, (**d**) ultimate rostral segment (URS), ventral side, (**e**) URS, dorsal side, (**f**) URS, lateral side, with trichoid sensilla forming three pairs of primary setae (dotted arrow) and trichoid sensilla as accessory setae (solid arrow), (**g**) surface of third rostral segment with flat denticles, (**h**) long trichoid sensillum, (**i**) short trichoid sensillum.

**Figure 7:**
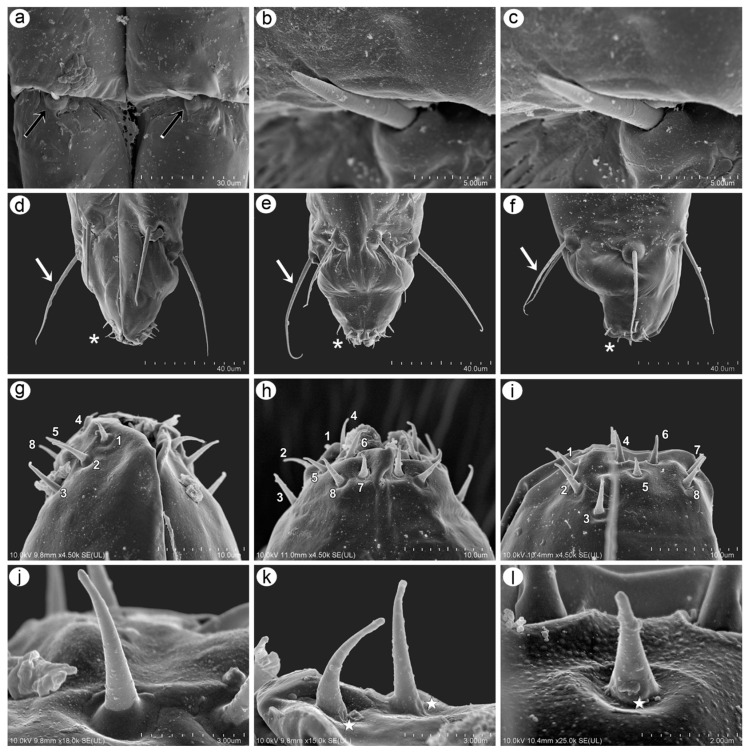
SEM images of sensilla on ultimate rostral segment: (**a**) proximal part of URS with type II basiconic sensilla (arrow), (**b**,**c**) ultrastructure of type II basiconic sensillum, (**d**) ventral apical part of IV+V with trichoid sensilla (arrow) and type III basiconic sensilla (asterisk), (**e**) dorsal apical part of IV+V with trichoid sensilla (arrow) and type III basiconic sensilla (asterisk), (**f**) lateral apical part of IV+V with trichoid sensilla (arrow) and type III basiconic sensilla (asterisk), (**g**) eight pairs of type III basiconic sensilla on apical end of IV+V seen from ventral side, (**h**) eight pairs of type III basiconic sensilla seen from dorsal side, (**i**) eight pairs of type III basiconic sensilla seen from lateral side, (**j**) ultrastructure of type III basiconic sensillum no. 1, (**k**) ultrastructure of type III basiconic sensilla no. 1 and no. 2 with visible molting pores (stars), (**l**) ultrastructure of type III basiconic sensillum no. 5 with visible molting pore (star), numbers in subfigures (**g**,**h**,**i**) indicates the sensilla pairs.

The mouthparts are clearly visible, especially from the ventral side of the body of the apterous viviparous female. On the ventral side of the head, in the central part, a large clypeus can be found and can be easily divided into the upper postclypeus and lower anteclypeus which passes into a flat and narrow labrum. The lateral sides of the head-connected mouthparts constitute clearly visible mandibular laminae and hidden small maxillary laminae (Figure 4d). The labial segments are characterized by long, fine, and pointed trichoid sensilla (Figure 6a–c). The first segment is short and membranous, and the rest of the segments are sclerotized. The second segment is the longest and is densely covered by numerous denticles, which form regular rows (Figure 6g,h). The rest of the segments are characterized by smooth cuticles. The third segment is slightly oval from the ventral side (Figure 6a), pear-shaped from the dorsal side (Figure 6b), and rectangular from the lateral view (Figure 6c). It bears four setae (trichoid) on the ventral side and four setae on the dorsal side (Figure 6a,b). The ultimate rostral segments (IV+V) are triangular from the dorsal and ventral sides (Figure 6a,b) and oval from the lateral view (Figure 6c). The ultimate rostral segments bear three kinds of sensilla: type I trichoid sensilla—three pairs on the border between the fourth and the fifth segment (primary setae) (Figure 7d–f) and all those on the fourth segment (accessory setae); two type II basiconic sensilla; and type III basiconic sensilla on the very apical part (Figure 6d–f). The type II basiconic sensilla are almost hidden in the border between segments III and IV. They are tubular and tapering, with pointed apices and spherical sockets (Figure 7a–c). The type III basiconic sensilla are located on the lateral and dorsal sides of the very apical part of the last segment, with eight pairs per each half (Figure 7g–i). Two lengths characterize the type III basiconic sensilla, and besides this difference, they are tubular, tapering with slightly rounded apices, and the molting pores are clearly visible (Figure 7j–l).

#### 3.2.4. Sensilla on Cuticle and Legs

**Figure 8:**
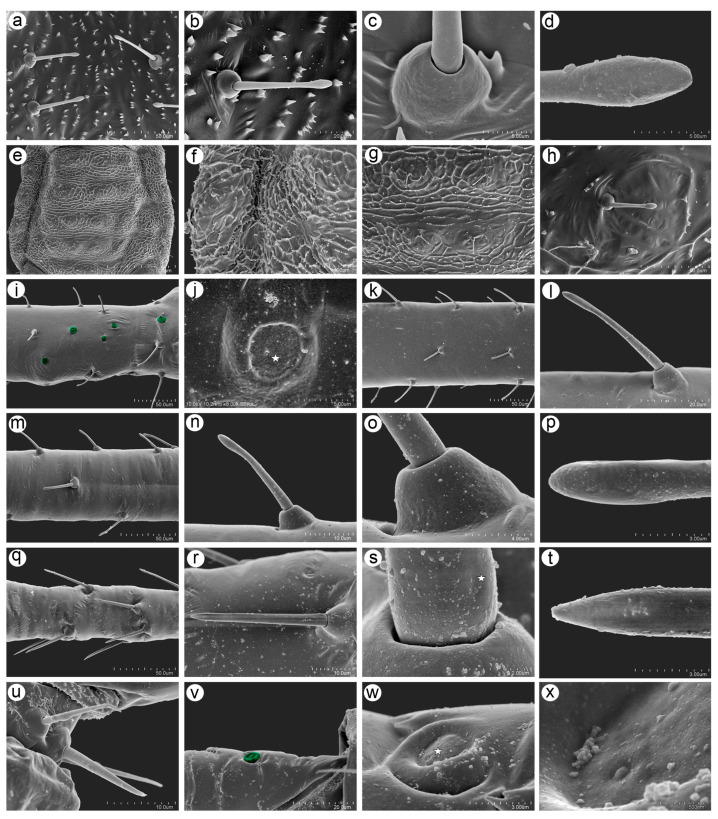
SEM images of sensilla on the body surface and sensilla on the legs of *Macromyzus* (*M*.) *diplazius* sp. nov.: (**a**) cuticle of the dorsal side of the head showing numerous denticles and trichoid sensilla with narrow-capitate apices, (**b**) ultrastructure of the trichoid sensillum of the head, (**c**) ultrastructure of the socket of the trichoid sensillum, (**d**) ultrastructure of the apical part of the trichoid sensillum, (**e**) dorsal side of the abdomen, (**f**) marginal area of the dorsal abdomen with numerous denticles forming polygonal sculptures, (**g**) spinal area of the dorsal abdomen with trichoid sensilla and polygonal sculpture, (**h**) ultrastructure of the trichoid sensillum, (**i**) inner side of the trochantro-femoral area with campaniform sensilla (green), (**j**) ultrastructure of the campaniform sensillum with a clearly visible pore (star), (**k**) trichoid sensilla with narrow capitate apices on hind femur (**l**) ultrastructure of femoral trichoid sensillum, (**m**) trichoid sensilla with narrow capitate apices on proximal and middle parts of hind tibia, (**n**) ultrastructure of the trichoid sensillum with narrow capitate apex, (**o**) ultrastructure of the socket of trichoid sensillum, (**p**) ultrastructure of the apex of trichoid sensillum, (**q**) trichoid sensilla with pointed apices on the distal part of hind femur, (**r**) ultrastructure of the trichoid sensillum with a pointed apex, (**s**) ultrastructure of the socket and basal part of trichoid sensillum with visible molting pore (star), (**t**) ultrastructure of the apex of the trichoid sensillum, (**u**) sensilla of the ventral side of the first segment of the hind tarsus, (**v**) campaniform sensillum (green) on the dorsal basal part of the second segment of the hind tarsus, (**w**,**x**) ultrastructure of the campaniform sensillum with a visible pore (star) and pore area.

The head cuticle is covered by numerous triangular denticles lying singly or in groups (2–6 denticles) and by type I trichoid sensilla (setae) which are tubular, long, rigid and with narrow capitate apices, 31–37 μm long (Figure 8a,b). The sockets of the sensilla are spherical or trapezoid with a clearly visible gap between the sensillum and socket edge (Figure 8c), and the apices are flat at the very end (Figure 8d). The dorsal cuticle on the thorax and abdomen is characterized by very clearly visible polygonal sculptures and low spinal protuberances (Figure 8e). The dorsal sculptures are denser in the pleural and marginal areas and are additionally covered by numerous denticles (Figure 8f), which are less numerous and more irregular in the spinal area (Figure 8g). The surrounding area of the spinal setae (trichoid sensilla) are poorly elevated and rather smooth, having only some denticles. The abdominal setae (trichoid sensilla) are shorter than those on the head, and their apices are also narrow capitate (Figure 8h). The legs are covered by campaniform sensilla and trichoid sensilla of different lengths and two types. Campaniform sensilla can be found on the inner side of the trochantera and proximal parts of the femora (Figure 8i). The campaniform sensilla are rounded, elevated, and 8.50–10.5 μm in diameter, and the inner plate is about 4.0–5.0 μm in diameter. The sensillum pore is located between the plate center and its lateral edge (Figure 8j). The trichoid sensilla on the femora are of different lengths, ranging from 20 to 25 μm in length, and are directed to the distal end (Figure 8k). The sensilla arise from trapezoid sockets. They are tubular in the basal and middle parts and slightly flattened in the apical part, which has a narrow capitate shape (Figure 8l). Similarly, the trichoid sensilla on the tibiae are oriented in two ways. From the proximal part of the tibia to the middle part, shorter sensilla can be found (Figure 8m). They are similar to the rest of the trichoid sensilla which are found on the antennae and dorsum, 20–31 μm long, tubular, rigid, and with narrow capitate apices (Figure 8n,p). The sockets of the sensilla are elevated, well developed, and trapezoid (Figure 8o). From the middle inner side and on the outer distal part of tibiae, much longer sensilla can be found (Figure 8q). They are 32–40 μm long, arise at an angle of 45°, and are straight, rigid, and with pointed apices (Figure 8r,t). Their sockets are also trapezoid, and sometimes, molting pores can be noted (Figure 8s). The trichoid sensilla on the first segment of the tarsi and two lateral setae are much thinner than the central one (Figure 8u). On the dorsal side of the proximal part of the hind tarsus, one campaniform sensillum is found (Figure 8v). The sensillum is circular with an robust outer collar, 3.80–5.00 μm in diameter, and the diameter of the inner plate is 1.70–2.80 μm with a clearly visible pore near to the edge of the plate (Figure 8w,x).

### 3.3. Key to Genus of Macromyzus Takahashi, 1960 (Apterous Viviparous Female)

#### 3.3.1. Key to Subgenera of *Macromyzus* Takahashi, 1960 (Apterous Viviparous Female)

1. ANT IV without secondary rhinaria; spot-shaped spinal sclerites on abdominal tergites with distinct tubercles………………...…..…*Macromyzus* Takahashi, 1960

- ANT IV with secondary rhinaria; band-shaped spinal sclerites on abdominal tergites without distinct tubercles............................ *Anthracosiphoniella* Basu, 1969

##### 3.3.2. Keys to Species of Subgenus *Macromyzus* Takahashi, 1960 (Apterous Viviparous Female) (Modified from [3,13])

1. ANT shorter than BL; PT 2.40–4.60 times as long as ANT VIb…………….. …………………………….………………..*M*. *manoji* Raha and Raychaudhuri, 1978

- ANT at least as long as or longer than BL; PT at least 5.00 times as long as ANT VIb………………...........................................................................................................2

2. Abdominal tergites without reticulations; PT 6.33–6.60 times as long as ANT VIb………………………………………….. *M*. *indicus* David and Narayanan, 1968

- Abdominal tergites with reticulations.........................................................................3

3. ANT III with 5–10 secondary rhinaria; URS 1.12–1.36 times longer than HT II.... …………………………………………………………….……….*M*. *diplazius* sp. nov

- ANT III without secondary rhinaria; URS more 1.40 times longer than HT II……4

4. URS 1.41–1.51 times longer than HT II; PT 6.08–6.43 times as long as ANT VIb.. …………………………………………………………*M*. *spinosus* Su and Qiao, 2010

- URS more 1.5 times longer than HT II; PT 5.19–6.31 times as long as ANT VIb... ……………………………………………………..*M*. *woodwardiae* (Takahashi, 1921)

## 4. Discussion

### 4.1. Taxonomical Comments

*Macromyzus* species have been reported in southeastern and eastern Asia, including China (Taiwan), India, Indonesia, Japan, Korea, and Nepal, and only five species have been identified to date [1,3]. This limited number of identified species may suggest low species diversity within the genus and a lack of comprehensive morphological information. Previous studies on *Macromyzus* have relied on limited morphological data for taxonomic research. According to Raychaudhuri [8] and Su and Qiao [3], the differentiation of subgenera within *Macromyzus* was based on morphological features, including the presence of secondary rhinaria on ANT III and ANT IV, as well as spinopleural patches on abdominal tergites with distinct tubercles. However, it is noteworthy that the new species possesses ANT III with secondary rhinaria and spot-shaped spinal sclerites on abdominal tergites bearing distinct tubercles. This unique combination of characteristics complicates the clear classification of the new species within the subgenera framework proposed by Raychaudhuri [8] and Su and Qiao [3]. Therefore, we have revised the taxonomical key for *Macromyzus*, using the morphology of secondary rhinaria on ANT IV, spinal sclerites on abdominal tergites, and the presence or absence of tubercles as more definitive criteria for subgenera differentiation. Through this reassessment, we have confirmed that the newly discovered species belongs to the subgenus *Macromyzus*.

### 4.2. Scanning Electron Microscopy of Sensilla

Aphids are herbivorous insects with a wide host range, including bryophytes, pteridophytes, gymnosperms, and angiosperms [1]. The evolution of their unique traits is intricately linked to their ecological relationships and co-diversification with host plants [14,15,16]. However, host adaptation, coevolutionary relationships, and detailed classification across taxa remain poorly understood. We propose that using scanning electron microscopy (SEM) on aphid sensory organs could help bridge this gap by providing essential insights for these studies. SEM has contributed to a deeper understanding of the morphological traits used in species identification and classification within the studied groups [17]. This study offers the first detailed morphological descriptions of the sensory organs in *Macromyzus*, a fern-feeding aphid genus. By examining antennal, mouthpart, and leg sensilla, we provide ultrastructural insights that can serve as valuable indicators for studying the coevolutionary relationships and phylogeny of aphids and their host plants.

## 5. Conclusions

In this study, we discovered a new species of *Macromyzus* in Laos and contributed to resolving taxonomic issues of previously understudied *Macromyzus* species through a highly detailed morphological analysis. Additionally, we established the taxonomic position of the new species by providing a comprehensive taxonomic key for all *Macromyzus* species worldwide. As the morphological study of aphid sensory organs is essential for understanding functional adaptations, future research should focus on other aphid taxa to better interpret the coevolution between aphids and their host plants.

## Figures and Tables

**Table 1:** Biometric data of *Macromyzus* (*M*.) *diplazius* sp. nov. (in mm).

Character	Apterous Viviparous Females (n = 12) (Mean, Range)
BL	2.68 (2.24–2.98)
BW	1.68 (1.36–1.85)
ANT III	0.85 (0.79–0.91)
ANT IV	0.63 (0.57–0.68)
ANT V	0.47 (0.44–0.49)
ANT VIb	0.14 (0.12–0.15)
PT	0.83 (0.75–0.92)
URS	0.153 (0.15–0.16)
BW URS	0.07 (0.06–0.08)
H FEM	1.14 (1.03–1.22)
H TIB	2.00 (1.74–2.22)
HT I	0.052 (0.050–0.055)
HT II	0.13 (0.12–0.14)
SIPH	0.95 (0.81–1.03)
BW SIPH	0.19 (0.15–0.23)
DW SIPH	0.061 (0.056–0.065)
Cauda	0.17 (0.15–0.19)
BW Cauda	0.15 (0.14–0.17)
BD ANT III	0.036 (0.034–0.038)
MW H TIB	0.047 (0.044–0.051)
Cephalic setae	0.05 (0.04–0.06)
Setae on ANT III	0.025 (0.023–0.028)
Setae on ABDT I	0.039 (0.035–0.042)
Setae on ABDT VIII	0.06 (0.05–0.07)
Setae on H TIB	0.036 (0.033–0.044)

## Data Availability

All data generated or analyzed during this study are included in this published article.
